# The Age-Dependent Increase of Metabolic Syndrome Requires More Extensive and Aggressive Non-Pharmacological and Pharmacological Interventions: A Cross-Sectional Study in an Italian Cohort of Obese Women

**DOI:** 10.1155/2021/5576286

**Published:** 2021-04-23

**Authors:** Antonello E. Rigamonti, Sabrina Cicolini, Sofia Tamini, Diana Caroli, Silvano G. Cella, Alessandro Sartorio

**Affiliations:** ^1^University of Milan, Department of Clinical Sciences and Community Health, Milan 20129, Italy; ^2^Instituto Auxologico Italiano, IRCCS, Experimental Laboratory for Auxo-endocrinological Research, Piancavallo (VB) 28824, Italy; ^3^Istituto Auxologico Italiano, IRCCS, Division of Auxology and Metabolic Diseases, Piancavallo (VB) 28824, Italy

## Abstract

**Background:**

Metabolic syndrome is a combination of cardiovascular risk factors (i.e., visceral obesity, dyslipidaemia, glucose intolerance, and hypertension), which entails critical issues in terms of medical management and public health.

**Methods:**

The aim of the present cross-sectional study was to investigate the age-related changes of the single IDF (International Diabetes Federation) diagnostic criteria for metabolic syndrome (waist circumference, WC; high-density lipoprotein cholesterol, HDL-C; triglycerides; glucose; systolic and diastolic blood pressure, SBP and DBP) in a large population of (Italian) obese women (*n* = 1.000; body mass index, BMI >30 kg/m^2^; age: 18–83 yrs), subdivided into two subgroups depending on the presence (*n* = 630) or absence (*n* = 370) of metabolic syndrome. Parallelly, the percentages of treatment with hypolipidaemic drugs, hypoglycaemics, and antihypertensives and, among the treated subjects, of control of the underlying condition in accordance with the cut-offs of IDF criteria for dyslipidaemia, hyperglycaemia, and hypertension were determined over six age ranges (i.e., 18–30, 31–40, 41–50, 51–60, 61–70, and > 70 yrs).

**Results:**

The prevalence of metabolic syndrome increased with advancing age. In the subgroup with metabolic syndrome, an age-dependent increase in HDL-C, glycaemia, and SBP occurred, while the visceral adiposity was stable. In the same subgroup, triglycerides and DBP decreased age-dependently. In the subgroup without metabolic syndrome, an age-dependent increase in WC, HDL-C, glycaemia, SBP, and DBP was observed. A progressive age-dependent increase in the percentage of patients pharmacologically treated for the cardiometabolic abnormalities was detected in patients with metabolic syndrome, a similar trend being also observed in patients without metabolic syndrome only for the antihypertensives. A clear-cut disproportion between treated versus adequately controlled women (with pharmacotherapy) was detected in the whole population.

**Conclusions:**

At least in an Italian context of obese females, the age-dependent worsening of glycaemia and BP exerts a fundamental pathophysiological role in the progressive increase of metabolic syndrome with advancing age, which appears to be not adequately treated in a large part of obese subjects. The results of the present study might be useful for public health decision-makers for programming future more extensive and aggressive non-pharmacological and pharmacological interventions in the obese population.

## 1. Introduction

Metabolic syndrome is a combination of cardiovascular risk factors, including visceral obesity, dyslipidaemia, glucose intolerance, and hypertension [1].

Although the prevalence of obesity is pandemic [2], there is still scarce awareness/evidence of metabolic syndrome, which remains underdiagnosed, insufficiently treated, and unsuccessfully controlled [3, 4]. The long-term consequences to which an obese patient with (uncontrolled) metabolic syndrome is exposed are well known, including atherosclerosis, acute coronary syndrome, stroke, type 2 diabetes mellitus, non-alcoholic fatty liver disease, and kidney chronic disease [5].

Indeed, body weight reduction programs allow us to reduce visceral obesity, improve glucometabolic homeostasis, and control hypertriglyceridemia and hypertension [6–9]; moreover, effective pharmacological interventions are available to treat dyslipidaemia, hyperglycaemia, and hypertension in clinical practice [10].

One of the main reasons for this disheartening contradiction is the missing “holistic” view of any obese patient seeking for clinical help to lose body weight, which, in most patients' opinion, is primarily thought to be an “aesthetic” problem, without paying attention to the associated cardiometabolic comorbidities [11].

Metabolic syndrome is a condition evolving over time, with a progressive increase in the number of cardiovascular risk factors in parallel with the natural history of the obese state, characterized by low-grade chronic inflammation, accelerated aging, and metabolic dysregulation [12].

To the best of our knowledge, while epidemiological analysis of cardiovascular risk factors in large populations of children/adolescents and adults was fundamental to diagnostically define metabolic syndrome, few authors have separately and collectively investigated the age-dependent changes of IDF (International Diabetes Federation) diagnostic criteria for metabolic syndrome in large study populations [13, 14]. Furthermore, pharmacological management—i.e., treatment and control—of the metabolic syndrome might vary among IDF diagnostic criteria and age ranges. Knowing the age-related evolution of metabolic syndrome in specific ethnic groups or geographic areas (i.e., the prevalently Caucasian obese population residing in Italy) might improve the clinical management of the obese patient and guide public health decision-makers in future investments of health care [15].

Based on the previous premises, the aim of the present cross-sectional study was to collect demographic, clinical, and biochemical data from a huge number of obese women with or without metabolic syndrome and analyse the age-dependent changes of visceral obesity, dyslipidaemia, glycaemia, and blood pressure together with the prescription of specific pharmacological interventions, particularly hypolipidaemic, hypoglycaemic, and antihypertensive drugs and, within the treated group, the pharmacological control of the underlying condition (i.e., dyslipidaemia, hyperglycaemia, and hypertension).

Our hypothesis is that, in a specifically Italian cohort of obese women, metabolic syndrome can be underdiagnosed, insufficiently treated, and unsuccessfully controlled. Moreover, the age-dependent increase in dyslipidaemia, hyperglycaemia, and hypertension could not be associated with a parallel prescribing of effective pharmacological interventions.

## 2. Materials and Methods

### 2.1. Patients

From January 2017 to December 2019, a population of 1.000 obese women (age ≥ 18 yrs and body mass index, BMI > 30 kg/m^2^) was recruited at the Division of Metabolic Diseases, Istituto Auxologico Italiano, Piancavallo (VB), where they were hospitalized for a three-week multidisciplinary integrated body weight reduction program (BWRP), entailing hypocaloric diet, nutritional education, psychological counselling, and moderate physical activity.

For each participant, anthropometric and instrumental measurements, metabolic variables, and pharmacological anamnesis were collected (see below for more details). Metabolic syndrome was calculated by using the IDF criteria (see also below) [16]. A very small percentage (less than 5%) of the recruited obese subjects was aware to have a diagnosis of metabolic syndrome in the medical history or had any clinical document reporting a diagnosis of metabolic syndrome before hospitalization to our institution.

### 2.2. Anthropometric Measurements

A scale with a stadiometer was used to determine height and weight (Wunder Sa.Bi., WU150, Trezzo sull'Adda, Italy). Waist circumference (WC) was measured with a flexible tape in a standing position, halfway between the inferior margin of the ribs, and the superior border of the crista. Body composition was measured by bioimpedance analysis (Human-IM Scan, DS-Medigroup, Milan, Italy) after 20 min of supine resting and in accordance with the international guidelines [17]. BMI, fat mass (FM), free-fat mass (FFM), and body mass fat index [BMFI : BMI × FM (%) × WC (cm)] [18, 19] were determined in all subjects.

### 2.3. Metabolic Variables

Blood samples (about 10 mL) were collected at around 8 : 00 AM after an overnight fast at the beginning of the BWRP. High-density lipoprotein cholesterol (HDL-C), triglycerides, and glucose were measured.

Colorimetric enzymatic assays (Roche Diagnostics, Monza, Italy) were used to determine serum HDL-C and triglycerides levels. The sensitivities of the assays were 3.09 mg/dL (1 mg/dL = 0.03 mmol/L) and 8.85 mg/dL (1 mg/dL = 0.01 mmol/L), respectively.

Serum glucose level was measured by the glucose oxidase enzymatic method (Roche Diagnostics, Monza, Italy). The sensitivity of the method was 2 mg/dL (1 mg/dL = 0.06 mmol/L).

### 2.4. Evaluation of Blood Pressure

Blood pressure (BP) was measured on the right arm, using a sphygmomanometer with appropriate adult cuff size, with the subject in a seated position and relaxed condition. The procedure was repeated three times at 10 min intervals in between; the means of the three values for systolic BP (SBP) and diastolic BP (DBP) were recorded.

### 2.5. Definition of Metabolic Syndrome

According to the IDF criteria for the diagnosis of metabolic syndrome in adults [16], obese (female) patients were considered positive for the presence of metabolic syndrome if they had three or more of the following factors: (i) abdominal obesity (WC ≥ 80 cm), (ii) increased triglycerides: ≥150 mg/dL (1.7 mmol/L) or specific treatment for this lipid abnormality; (iii) reduced HDL-C: <50 mg/dL (1.3 mmol/L) or specific treatment for this lipid abnormality; (iv) increased BP : SBP ≥1 30 mmHg or DBP ≥ 85 mmHg and/or treatment of previously diagnosed hypertension; (v) increased fasting plasma glucose (FPG) concentration ≥100 mg/dL (5.6 mmol/L) or previously diagnosed type 2 diabetes mellitus.

### 2.6. Statistical Analysis

The Sigma Stat 3.5 statistical software package (Systat Software, San Jose, CA, USA) was used for data analyses.

Results are reported as mean ± SD (standard deviation) or percentage (as specified in tables) for six age ranges (i.e., 18–30, 31–40, 41–50, 51–60, 61–70, and > 70 yrs).

Before applying any parametric test, the normal distribution and linearity of each variable were verified, and a log-transformation of the variable was performed, if appropriate.

Some parameters, such as age, BMI, BMFI, FM, FFM, WC, HDL-C, triglycerides, glucose, and BP, were compared between obese women with or without metabolic syndrome by using a *t*-student test for unpaired data. Other parameters, such as percentages of subjects positive/negative for metabolic syndrome and for each altered IDF diagnostic criteria, of treated patients (hypolipidaemic drugs, hypoglycaemics, and antihypertensives) and, within the treated groups, of controlled subjects (in accordance with the cut-offs of IDF diagnostic criteria for metabolic syndrome) were compared between obese women with or without metabolic syndrome by using a Fisher's exact test.

Furthermore, a model of linear regression was used to analyse the age-dependence of each of the above-reported parameters.

A level of significance of *p* < 0.05 was used for all data analyses.

## 3. Results

Using the IDF criteria for the definition of metabolic syndrome, 630 (63.0%) had metabolic syndrome and 370 were without (37.0%).

A significant age-dependent increase in the prevalence of metabolic syndrome occurred (from 25.5% in the age range 18–30 yrs up to 71.0% in the range over 70 yrs). BMI and BMFI significantly decreased with advancing age in obese women with metabolic syndrome; this pattern was not recorded in those without metabolic syndrome. FM (%) and FFM (kg) decreased over time in both groups with and without metabolic syndrome (Table 1).

BMI was significantly higher in obese women with metabolic syndrome than in those without metabolic syndrome in the age ranges 31–40 and 51–60 yrs, while BMFI was significantly higher in obese women with metabolic syndrome aged 31–60 yrs when compared with the corresponding subgroup without metabolic syndrome. Only in the perimenopausal period, FM (%) and FFM (kg) were significantly higher in the group with metabolic syndrome than that without metabolic syndrome (Table 1).

WC significantly increased with advancing age only in obese women without metabolic syndrome, being an anthropometric parameter stable over time in the group with metabolic syndrome. While a significant age-dependent increase in HDL-C occurred in both groups with and without metabolic syndrome, triglycerides significantly decreased with advancing age only in obese women with metabolic syndrome. A significant worsening in glycaemia occurred over time in obese women independently of the diagnosis of metabolic syndrome. SBP significantly increased with advancing age in both patients with and without metabolic syndrome. By contrast, DBP increased with advancing age in patients without metabolic syndrome, while it reduced in patients with metabolic syndrome. An increase in the total score of altered IDF criteria for metabolic syndrome occurred age-dependently in both groups with and without metabolic syndrome (Table 2).

HDL-C levels were significantly higher in patients without metabolic syndrome than in those with metabolic syndrome in all age subgroups, while WC (up to the sixth decade), triglycerides, and glucose were significantly higher in obese women with metabolic syndrome than in those without metabolic syndrome. SBP was significantly higher in obese women with metabolic syndrome than in those without metabolic syndrome up to the fifth decade, while DBP up to the fourth decade. Finally, the total score of altered IDF criteria was significantly higher in the group with metabolic syndrome than in that without metabolic syndrome in all age ranges (Table 2).

Altered WC (ie ≥ 80 cm) was present in all subjects. The percentage of obese women with HDL-C <50 mg/dl significantly decreased with advancing age in both patients with and without metabolic syndrome. The percentage of obese women with triglycerides ≥150 mg/dl remained stable with advancing age in both patients with and without metabolic syndrome. The percentage of obese women with glucose ≥100 mg/dl significantly increased with advancing age only in the group with metabolic syndrome, the peak being reached in the age range >70 yrs, while no age-dependent changes were recorded in patients without metabolic syndrome. The percentage of obese women with SBP ≥130 mmHg and/or DBP ≥85 mmHg significantly increased in both groups, the peak being reached at 61–70 yrs in patients with metabolic syndrome and >70 yrs in those without metabolic syndrome (Table 3).

The percentages of obese women with altered values of HDL-C, triglycerides, glucose, and BP (in accordance with the cut-offs of IDF criteria) were significantly higher in the group with metabolic syndrome than without metabolic syndrome, the unique exception being represented by BP (age range >70 yrs) which was comparable between patients with and without metabolic syndrome (Table 3).

A progressive age-dependent increase in the percentage of patients pharmacologically treated for the abnormalities concurring to determinate metabolic syndrome was detected in patients with metabolic syndrome (age range 18–30 yrs versus age range >70 yrs; hypolipidaemic drugs: from 8% up to 22.6%; hypoglycaemics: from 12.0% up to 40.8%; antihypertensives: from 28.0% up to 87.8%), a similar trend being also observed in patients without metabolic syndrome only for the antihypertensives (from 1.4% up to 70.0%) (Table 4).

Analysing the data of the treated groups, despite a significant age-dependent increase in the percentages of treated patients, a clear-cut disproportion was found between treated and adequately controlled (with pharmacotherapy) patients in the whole population (23.4% for glycaemia and 44.8% for BP), the inadequate treatment being present in all the age ranges. The only exception was in pharmacological control of hypertriglyceridemia, which increased with advancing age (Table 5).

## 4. Discussion

The main results of the present cross-sectional study, carried out in a huge number of (Italian) obese women with or without metabolic syndrome, aged from 18 to 83 yrs, can be summarised in the following points:

1. The prevalence of metabolic syndrome increases with advancing age (from 25.5% in the age range 18–30 yrs up to 71.0% in patients over 70 yrs), being the age-dependent worsening of glucose and BP control the pathophysiological driver of its increase
2. IN the group without metabolic syndrome, an age-dependent increase in WC, glycaemia, SBP, and DBP was observed, indicating a progressive overall cardiometabolic deterioration
3. A progressive age-dependent increase in the percentage of patients pharmacologically treated for the abnormalities concurring to determinate metabolic syndrome is detected in patients with metabolic syndrome (age range 18–30 yrs versus age range over 70 yrs; hypolipidaemic drugs: from 8% up to 22.6%; hypoglycaemics: from 12.0% up to 40.8%; antihypertensives: from 28.0% up to 87.8%), a similar trend being also observed in patients without metabolic syndrome only for the antihypertensives (from 1.4% up to 70.0%)
4. Surprisingly, among the pharmacologically treated patients, only a relatively small percentage of obese women (particularly within the group with metabolic syndrome) is successfully controlled in accordance with the cut-offs of IDF criteria (for dyslipidemia, glycaemia, and hypertension)
5. A clear-cut disproportion between treated versus adequately controlled (with pharmacotherapy) patients is detected (adequately controlled in 23.4% with hypoglycaemics and 44.8% with antihypertensives in the whole population)

The age-dependent worsening of hyperglycaemia in obese patients (with or without type 2 diabetes mellitus), as demonstrated also in the present study, is related to the well-known progressive age-dependent acceleration of *β*-cell dysfunction [20]. Paradoxically, in the present study, WC increased only in women without metabolic syndrome, while BMI decreased in those with metabolic syndrome with the age advancement; the first one probably depends upon the progressive worsening of obesity in those patients less compromised and less careful to their conditions, and the second one is probably related to the greater awareness of their disease in those heavier and with metabolic syndrome. Anyway, in metabolic syndrome, adipose tissue, mainly that located in the visceral compartment, is phenotypically inflamed, with release of adipokines/cytokines and spill-over of toxic lipids which, altogether, derange glucometabolic homeostasis [21]. In particular, triglycerides decrease with advancing age in our patients with metabolic syndrome, being always higher in the group with metabolic syndrome than without metabolic syndrome in all age ranges.

As shown in the present study, the potentially cardioprotective age-dependent increase of HDL-C in both groups with and without metabolic syndrome is of difficult interpretation. In fact, after the perimenopausal period, a reduction of the protective oestrogen effect on cholesterol is usually reported in normal weighted subjects [22]. Since the age-dependent pattern of HDL-C is common to both obese women with and without metabolic syndrome, the former having lower values than the latter, it can be hypothesized that the lack of HDL-C reduction after menopause is a peculiarity of obese patients in this phase of life [23, 24].

In the present study, the age-dependent SBP increase was comparable in patients with and without metabolic syndrome, although the latter started from higher values and were more frequently treated. A different age-dependent pattern was observed for DBP, a progressive reduction being found in patients with metabolic syndrome and an increase in those without. Although our interpretation may be speculative because of the missing normal-weight group as a control, atherosclerosis, endothelial dysfunction, and sympathetic hyperactivation are likely to play a relevant pathophysiological role in SBP increase with advancing age in obese women, more evidently in the group with metabolic syndrome than without metabolic syndrome [25].

To the best of our knowledge, few authors have evidenced in a single study an astonishing contradiction between the natural history of metabolic syndrome, i.e., an age-dependent increase in the prevalence, and its pharmacological management, i.e., insufficient/unsuccessful control [26].

Due to the methodological limitations of the present study, we can only tentatively explain the reasons for this evidence-based contradiction, which will require a confirmation from long-term prospective studies.

First of all, in view of the unsuccessful control of obese women with metabolic syndrome, a “holistic” approach should be adopted in clinical practice when an obese patient comes to medical attention willing to lose body weight. Dyslipidemia, glucose intolerance, and hypertension should be strenuously sought, pharmacologically treated, and successfully controlled [11]. This “holistic” approach should be preferentially adopted by the general practitioner, who, in the Italian context, represents the so-called primary care [27].

Nevertheless, as demonstrated by the present study carried out in obese women seeking hospitalization in a third-level setting for obesity, the prescription of pharmacotherapy for all the abnormalities involved in the determination of the metabolic syndrome, such as dyslipidemia, hyperglycaemia, and hypertension, appears seriously insufficient and unsuccessful. This may depend on a difficulty by the general practitioner to assist obese patients (mainly those more compromised) or to manage hypoglycaemic and antihypertensive drugs (particularly when their combination is required) in severely obese patients [28]. The observed undertreatment could be also explained by the poor compliance of obese patients, who frequently underestimate their clinical condition and do not adhere to the medical prescriptions. In this regard, the present study shows that undertreatment, particularly with hypoglycaemics, predominantly involved older obese women. Although hypoglycaemia may be actually a serious side effect of hypoglycaemics in the geriatric population, this should not be the reason for excluding older people from adequate treatments [29].

Medical management of metabolic syndrome does not include only pharmacological interventions. In fact, the administration of a BWRP to obese patients has been demonstrated to be extremely effective in improving most of the cardiometabolic conditions underlying the metabolic syndrome, such as dyslipidemia, glucose intolerance, and hypertension [7, 8]. In this respect, the early admission of obese patients to a BWRP (third level of health care) might be a valid solution of public health to interrupt the vicious circle referring to the natural history of metabolic syndrome [30] and the serious issues of underdiagnosis/undertreatment, which have been evidenced in the present study.

Taking into account the contradiction between high prevalence and scarce control of metabolic syndrome, public health decision-makers are asked for the development of more effective strategies able to fight against obesity and cardiometabolic comorbidities [15].

Before closing, some limitations of the present study should be mentioned.

Although not all the subjects were successfully controlled, previous and current treatments may have certainly influenced the results of this study. In particular, the use of hypolipidaemic drugs over time could partly explain an increase in HDL-C and a decrease in triglycerides with advancing age. Moreover, the higher prevalence of treated patients with metabolic syndrome could have reduced the difference between both groups for several parameters. Anyway, due to the limitation of any clinical study with a cross-sectional design, it is statistically difficult to isolate and weigh the covariate “treatment”. Unfortunately, we do not know exactly when any treatment (with hypolipidaemic, hypoglycaemic, and antihypertensive drugs) has been started and, importantly, maintained over time.

Apart from the cross-sectional design, which has been already highlighted above, drugs prescribed to patients (before admission to our institution) were generically classified as hypolipidaemic drugs, hypoglycaemics, and antihypertensives. Therefore, we cannot rule out that some specific pharmacological classes have been more frequently prescribed and/or could have more successfully controlled the underlying condition. Furthermore, the “correct” drug prescription by a general practitioner does not mean a “correct” drug intake by a patient due to problems of pharmacological adherence [31]. Moreover, our analysis did not take into consideration the duration of the single treatments, information not requested for the determination of metabolic syndrome based on cut-off values or specific treatment for the abnormalities (HDL-C, glycaemia, triglycerides, and BP) [15].

Hypolipidaemic drugs prescribed in clinical practice (such as statins) are more effective in reducing total and LDL cholesterol rather than in elevating HDL-C [32]. Anyway, being HDL-C one of the five IDF criteria determining metabolic syndrome [16], we have preferred to include this biochemical parameter in the statistical analysis.

In the present study, the effectiveness of any pharmacological treatment for dyslipidaemia, glucose intolerance, and hypertension was evaluated in accordance with the cut-offs of the IDF criteria [16], which fundamentally serve as a diagnostic tool for metabolic syndrome and might be very restrictive when adopted as therapeutic targets in clinical practice [33]. Nevertheless, the percentage of obese patients with insufficient control of the parameters determining metabolic syndrome found in the present study is so relevant that it seems plausible it can be poorly modified even considering less ambitious therapeutic targets.

Finally, the present study was carried out in the Italian context, encompassing an obese female population with specific gender-related, demographic, anthropometric, socioeconomic, and cultural/behavioural characteristics that may be different from those of another obese population living in other European or extra-European countries. Similar considerations are valid for the national health systems, which, even among close countries within the European community, may be extremely different. Finally, subjects included in the current study might also be non-representative of other Italian obese women issued from the same population because they were recruited among women willing to be hospitalized for a 3-week BWRP. Such program is highly demanding and includes highly motivated subjects, which could have influenced the results (e.g., treatment compliance). Therefore, based on the previous considerations, caution is necessary before extending the results of the present study in other (Italian or non-Italian) contexts.

## 5. Conclusions

At least in an Italian context, glucose intolerance and systolic hypertension play a fundamental pathophysiological role in the natural history of metabolic syndrome in adulthood, and the contributions of visceral adiposity and dyslipidaemia with advancing age were less relevant. The single components determining metabolic syndrome appear to be poorly controlled. The results of the present study might be useful for public health decision-makers for programming future more extensive and aggressive non-pharmacological and pharmacological interventions in the obese population, aimed to obtain better control of the alterations determining metabolic syndrome (and the risk for cardiovascular diseases) during the different periods of adult and elderly life.

## Figures and Tables

**Table 1:** Demographic characteristics and body composition in the entire obese population of our study and in the patients with or without metabolic syndrome, subdivided among age ranges.

Parameter	18–30	31–40	41–50	51–60	61–70	>70	*β*1	*Y*-intercept	*p*
*Number* (n.)									
** **ALL patients	98	106	196	292	239	69	—	—	—
** **MetS+	25 (25.5%)	54 (50.9%)	117 (59.7%)	203 (69.5%)	182 (76.2%)	49 (71.0%)	0.0098	0.12	<0.001
** **MetS-	73 (74.5%)	52 (49.1%)	79 (40.3%)	89 (30.5%)	57 (23.8%)	20 (29.0%)	—	—	—

*Age* (yr)									
** **ALL patients	24.7 ± 3.5	36.4 ± 2.8	46.0 ± 2.8	55.6 ± 3.0	65.1 ± 2.8	73.9 ± 2.6	—	—	—
** **MetS+	26.0 ± 3.2	36.9 ± 2.5	46.2 ± 2.8	55.4 ± 3.1	65.1 ± 2.8	74.0 ± 2.5	—	—	—
** **MetS-	24.3 ± 3.6	35.9 ± 2.9	45.7 ± 2.8	56.0 ± 2.7	65.3 ± 3.0	73.8 ± 2.8	—	—	—

*BMI* (kg/m^2^)									
** **ALL patients	44.3 ± 7.5	44.5 ± 6.9	43.6 ± 6.4	43.9 ± 6.2	43.6 ± 5.7	41.8 ± 4.6	−0.0289	45.23	0.044
** **MetS+	46.2 ± 11.6	46.0 ± 8.0^a^	44.1 ± 7.2	44.9 ± 6.5^a^	43.6 ± 5.7	41.7 ± 4.6	−0.0745	48.36	0.001
** **MetS-	43.6 ± 5.3	43.0 ± 5.2	42.8 ± 5.1	41.8 ± 5.0	43.6 ± 5.9	42.2 ± 4.7	−0.0185	43.69	0.295

*BMFI*									
** **ALL patients	27.7 ± 8.9	29.0 ± 10.6	27.5 ± 9.6	27.9 ± 9.4	27.5 ± 8.6	24.9 ± 5.4	−0.0328	29.34	0.1411
** **MetS+	27.9 ± 10.6	32.1 ± 12.2^a^	28.9 ± 10.8^a^	29.8 ± 10.0^a^	27.5 ± 8.4	24.9 ± 5.2	−0.0971	34.06	0.006
** **MetS-	27.6 ± 8.3	26.1 ± 7.8	25.6 ± 7.5	24.0 ± 6.2	27.5 ± 9.2	24.9 ± 6.2	−0.0375	27.66	0.175

*FM (%)*									
** **ALL patients	53.1 ± 5.3	52.8 ± 5.8	51.2 ± 5.0	50.7 ± 5.5	49.8 ± 5.4	48.3 ± 4.4	−0.0890	55.51	<0.001
** **MetS+	52.3 ± 5.1	53.7 ± 6.6	51.6 ± 5.2	51.5 ± 5.5^a^	49.6 ± 5.1	48.0 ± 4.1	−0.1075	56.85	<0.001
** **MetS-	53.4 ± 5.4	51.9 ± 4.8	50.5 ± 4.8	48.9 ± 4.8	50.6 ± 6.0	49.0 ± 5.2	−0.0895	55.01	<0.001

*FFM* (kg)									
** **ALL patients	52.4 ± 5.7	52.2 ± 4.8	53.0 ± 4.5	52.0 ± 5.0	51.8 ± 4.6	50.6 ± 3.8	−0.0370	54.17	0.002
** **MetS+	52.3 ± 6.4	54.1 ± 5.2	53.7 ± 4.9^a^	52.6 ± 5.3^a^	52.0 ± 4.7	51.0 ± 3.9	−0.05917	55.87	0.001
** **MetS-	52.5 ± 5.5	52.4 ± 3.8	52.1 ± 3.7	50.8 ± 3.9	51.2 ± 4.5	49.6 ± 3.5	−0.0433	53.68	0.005

Abbreviations: BMI, body mass index; BMFI, body mass fat index: BMI × WC × FM%; FFM, fat-free mass; FM, fat mass; MetS-, negative for metabolic syndrome; MetS+, positive for metabolic syndrome. ^a^: *p* < 0.05 versus the corresponding MetS- subgroup (Student's *t*-test for unpaired data). Values of *β*_1_, *Y*-intercept, and *p* refer to the linear regression performed by using all data within each subgroup (MetS+ or MetS-) or the entire population (MetS+/MetS-). The symbol “-“ means non-determinable or non-applicable.

**Table 2:** Values of the parameters adopted as criteria by IDF for the diagnosis of metabolic syndrome (waist circumference, dyslipidemia, hyperglycaemia, and hypertension) and number of the altered parameters in accordance with the cut-offs established by IDF in the entire obese population of our study and in the patients with or without metabolic syndrome, subdivided among age ranges.

IDF parameter	18–30	31–40	41–50	51–60	61–70	>70	*β*1	*Y*-intercept	*p*
*WC* (cm)									
** **ALL patients	118.1 ± 16.1	119.5 ± 13.2	119.7 ± 12.2	122.0 ± 12.6	123.2 ± 11.3	120.6 ± 9.4	0.1029	115.70	<0.001
** **MetS+	124.0 ± 22.7^a^	124.3 ± 13.0^a^	122.3 ± 13.1^a^	124.5 ± 12.4^a^	123.7 ± 11.0	120.9 ± 8.8	−0.0209	124.70	0.620
** **MetS-	116.1 ± 12.7	114.5 ± 11.5	115.8 ± 11.3	116.3 ± 11.3	121.6 ± 12.0	120.0 ± 10.8	0.1038	112.00	0.009

*HDL-C* (mg/dl)									
** **ALL patients	47.6 ± 11.2	46.4 ± 12.8	48.3 ± 11.9	51.2 ± 12.7	51.1 ± 12.5	51.2 ± 12.4	0.1232	43.31	<0.001
** **MetS+	41.7 ± 7.7^a^	40.6 ± 9.5^a^	43.3 ± 8.6^a^	47.1 ± 11.4^a^	47.8 ± 11.7^a^	47.6 ± 12.1^a^	0.2058	34.51	<0.001
** **MetS-	49.6 ± 11.6	52.5 ± 12.9	55.7 ± 12.1	60.4 ± 10.5	61.6 ± 8.4	60.1 ± 8.3	0.2843	42.94	<0.001

*Triglycerides* (mg/dl)									
** **ALL patients	108.8 ± 62.5	133.1 ± 75.0	134.4 ± 65.1	138.9 ± 64.8	134.7 ± 61.1	131.9 ± 57.5	0.3873	112.8	0.009
** **MetS+	159.6 ± 96.4^a^	164.8 ± 89.6^a^	158.9 ± 70.1^a^	158.0 ± 67.1^a^	145.8 ± 63.5^a^	145.0 ± 62.2^a^	−0.4924	181.4	0.036
** **MetS-	91.4 ± 30.9	100.2 ± 32.5	98.3 ± 32.5	95.5 ± 28.4	99.2 ± 34.3	100.0 ± 22.9	0.1148	91.34	0.272

*Glucose* (mg/dl)									
** **ALL patients	79.0 ± 19.8	86.5 ± 21.8	95.2 ± 30.8	102.6 ± 33.5	104.1 ± 30.4	106.9 ± 35.0	0.6070	66.06	<0.001
** **MetS+	89.3 ± 35.0^a^	93.4 ± 28.0^a^	102.8 ± 37.4^a^	111.0 ± 36.8^a^	108.9 ± 32.4^a^	114.9 ± 38.1^a^	0.5345	77.30	<0.001
** **MetS-	75.5 ± 8.5	79.5 ± 8.2	83.9 ± 8.4	83.5 ± 7.8	88.7 ± 15.2	85.8 ± 9.0	0.2632	69.94	<0.001

*SBP* (mmHg)									
** **ALL patients	119.6 ± 10.8	123.9 ± 11.4	125.5 ± 14.0	128.7 ± 13.7	132.0 ± 15.5	131.0 ± 16.1	0.2724	113.40	<0.001
** **MetS+	129.4 ± 12.4^a^	127.0 ± 11.8^a^	127.1 ± 13.7^a^	129.6 ± 14.0	131.7 ± 13.8	130.0 ± 15.8	0.1166	123.10	0.011
** **MetS-	116.2 ± 7.8	120.7 ± 10.0	123.0 ± 14.1	126.7 ± 13.1	132.7 ± 20.1	133.5 ± 16.9	0.3643	107.10	<0.001

*DBP* (mmHg)									
** **ALL patients	74.3 ± 8.3	77.5 ± 8.6	76.3 ± 8.0	77.7 ± 7.3	77.6 ± 7.4	75.8 ± 9.1	0.0449	74.55	0.013
** **MetS+	79.8 ± 9.2^a^	79.3 ± 8.3^a^	76.7 ± 7.9	77.7 ± 7.5	77.3 ± 7.0	75.3 ± 8.7	−0.0507	80.24	0.050
** **MetS-	72.4 ± 7.2	75.7 ± 8.6	75.6 ± 8.1	77.5 ± 6.7	78.5 ± 8.6	77.0 ± 9.9	0.1173	70.44	<0.001

*Altered parameters* (n)									
** **ALL patients	2.1 ± 1.0	2.6 ± 1.1	2.9 ± 1.1	3.2 ± 1.2	3.4 ± 1.2	3.3 ± 1.1	0.0265	1.63	<0.001
** **MetS+	3.5 ± 0.7^a^	3.5 ± 0.8^a^	3.6 ± 0.7^a^	3.8 ± 0.8^a^	3.9 ± 0.8^a^	3.8 ± 0.8^a^	0.0107	3.17	<0.001
** **MetS-	1.6 ± 0.5	1.7 ± 0.5	1.7 ± 0.4	1.8 ± 0.4	1.8 ± 0.4	1.9 ± 0.3	0.0037	1.55	0.013

Abbreviations: DBP, diastolic blood pressure; HDL-C, high-density lipoprotein cholesterol; MetS-, negative for metabolic syndrome; MetS+, positive for metabolic syndrome; SBP, systolic blood pressure; WC, waist circumference. a: *p* < 0.05 versus the corresponding MetS- subgroup (Student's *t*-test for unpaired data). Values of *β*1, *Y*-intercept, and *p* refer to the linear regression performed by using all data within each subgroup (MetS+ or MetS−) or the entire population (MetS+/MetS−).

**Table 3:** Obese patients with or without metabolic syndrome, subdivided among age ranges, having altered IDF criteria.

Evidence									
IDF criteria (cut-off)	18–30 (*n* = 98)	31–40 (*n* = 106)	41–50 (*n* = 196)	51–60 (*n* = 292)	61–70 (*n* = 239)	>70 (*n* = 69)	*β*_1_	Y-Intercept	*p*
*WC* (≥80 cm)									
** **MetS+	25 (100%)	54 (100%)	117 (100%)	203 (100%)	182 (100%)	49 (100%)	—	—	—
** **MetS−	73 (100%)	52 (100%)	79 (100%)	89 (100%)	57 (100%)	20 (100%)	—	—	—

*HDL-C* (<50 mg/dl)									
** **MetS+	24 (96%)^a^	50 (92.6%)^a^	104 (88.9%)^a^	146 (71, 9%)^a^	138 (75.8%)^a^	35 (71.4%)^a^	−0.0061	1.12	<0.001
** **MetS−	39 (53.4%)	20 (38.5%)	18 (22.8%)	6 (6.7%)	4 (7.0%)	0 (0.0%)	−0.0121	0.80	<0.001

*Triglycerides* (≥150 mg/dl)									
** **MetS+	13 (52%)^a^	33 (61.1%)^a^	59 (50.4%)^a^	117 (57.6%)^a^	93 (51.1%)^a^	26 (53.1%)^a^	−0.0005	0.57	0.745
** **MetS−	2 (2.7%)	1 (1.9%)	4 (5.1%)	2 (2.2%)	1 (1.7%)	0 (0.0%)	−0.0003	0.04	0.559

*Glucose* (≥100 mg/dl)									
** **MetS+	7 (28%)^a^	16 (29.6%)^a^	47 (40.2%)^a^	119 (58.6%)^a^	114 (62.6%)^a^	31 (63.3%)^a^	0.0104	−0.11	<0.001
** **MetS−	0 (0.0%)	0 (0.0%)	2 (2.5%)	3 (3.4%)	2 (3.5%)	0 (0.0%)	0.0006	−0.013	0.138

*BP* (SBP ≥130 and/or DBP≥85 mmHg)									
** **MetS+	18 (72%)^a^	37 (68.5%)^a^	97 (82.9%)^a^	192 (94.6%)^a^	176 (96.7%)^a^	47 (95.9%)	0.0069	0.52	<0.001
** **MetS−	6 (8.2%)	14 (26.9%)	34 (43.0%)	58 (65.2%)	36 (63.1%)	18 (90.0%)	0.0154	−0.28	<0.001

Abbreviations: DBP, diastolic blood pressure; HDL-C, high-density lipoprotein cholesterol; MetS-, negative for metabolic syndrome; MetS+, positive for metabolic syndrome; SBP, systolic blood pressure; WC, waist circumference. a: *p* < 0.05 versus the corresponding MetS- subgroup (Fisher's exact test). Values of *β*1, *Y*-intercept and *p* refer to the linear regression performed by using all data within each subgroup (MetS + or MetS-).

**Table 4:** Obese patients with or without metabolic syndrome, subdivided among age ranges, treated with hypolipidaemic drugs, hypoglycaemics, and/or antihypertensives.

	Treated								
	18–30	31–40	41–50	51–60	61–70	>70	*β*_1_	*Y*-intercept	*p*
Hypolipidaemics									
MetS+	2/25 (8.0%)	9/54 (16.7%)^a^	9/117 (7.7%)^a^	45/203 (22.2%)^a^	41/182 (22.5%)^a^	11/49 (22.4%)	0.0042	−0.05	0.001
MetS−	0/73 (0%)	0/52 (0%)	0/79 (0%)	0/89 (0%)	0/57 (0%)	0/20 (0%)	—	—	—

Hypoglycaemics									
MetS+	3/25 (12.0%)^a^	12/54 (22.2%)^a^	26/117 (22.2%)^a^	78/203 (38.4%)^a^	60/182 (33.0%)^a^	20/49 (40.8%)^a^	0.0057	−0.001	<0.001
MetS−	0/73 (0%)	0/52 (0%)	0/79 (0%)	1/89 (1.1%)	1/57 (1.8%)	0/20 (0%)	0.0002	−0.01	0.319

Antihypertensives									
MetS+	7/25 (28.0%)^a^	26/54 (48.1%)^a^	79/117 (67.5%)^a^	159/203 (78.3%)	155/182 (85.2%)^a^	43/49 (87.8%)	0.0115	0.11	<0.001
MetS−	1/73 (1.4%)	6/52 (11.5%)	22/79 (27.8%)	47/89 (52.8%)	28/57 (49.1%)	14/20 (70.0%)	0.0140	−0.34	<0.001

Values are expressed in percent (% to the number of patients within the corresponding age range). Abbreviations: MetS−, negative for metabolic syndrome; MetS+, positive for metabolic syndrome. ^a^*p* < 0.05 vs. the corresponding MetS− subgroup (Fisher's exact test). Values of β_1_, Y-intercept and p refer to the linear regression performed by using all data within each subgroup (MetS+ or MetS−). The symbol “—” means non-determinable or non-applicable.

**Table 5:** Obese patients with or without metabolic syndrome, subdivided among age ranges, treated with hypolipidaemic drugs, hypoglycaemics, and/or antihypertensives and successfully controlled in accordance with the cut-offs of the IDF diagnostic criteria for HDL-C, triglycerides, glucose, and blood pressure.

Successfully controlled									
IDF parameter	18–30	31–40	41–50	51–60	61–70	>70	*β*1	*Y*-intercept	*p*
*HDL-C* (≥50 mg/dl)									
** **MetS+	1/2 (50.0%)	1/9 (11.1%)	4/9 (44.4%)	14/45 (31.1%)	20/41 (48.8%)	4/11 (36.4%)	0.005	0.85	0.247
** **MetS-	—	—	—	—	—	—	—	—	—

*Triglycerides* (<150 mg/dl)									
** **MetS+	1/2 (50.0%)	5/9 (55.5%)	3/9 (33.3%)	17/45 (37.8%)	26/41 (63.4%)	8/11 (72.7%)	0.009	-0.022	0.038
** **MetS-	—	—	—	—	—	—	—	—	—

*Glucose* (<100 mg/dl)									
** **MetS+	2/3 (66.7%)	5/12 (41.6%)	4/26 (15.4%)	18/78 (23.1%)	15/60 (25.0%)	2/20 (10.0%)	−0.005	0.53	0.067
** **MetS-	—	—	—	1/1 (100.0%)	0/1 (0.0%)	—	—	—	—

*Blood pressure* (SBP <130 and/or DBP <85)									
** **MetS+	2/7 (28.6%)	12/26 (46.1%)	41/79 (51.9%)	73/159 (45.9%)	66/155 (42.6%)	21/43 (48.8%)	−0.001	0.53	0.551
** **MetS-	0/1 (0.0%)	2/6 (33.3%)	11/22 (50.0%)	21/47 (44.7%)	8/28 (28.6%)	6/14 (42.8%)	−0.003	0.61	0.425

Values are expressed in percent (% to the number of patients within the corresponding age range). Abbreviations: MetS-, negative for metabolic syndrome; MetS+, positive for metabolic syndrome. a: *p* < 0.05 versus the corresponding MetS- subgroup (Fisher's exact test). Values of *β*1, *Y*-intercept, and *p* refer to the linear regression performed by using all data within each subgroup (MetS + or MetS-). The symbol “-” means non-determinable or no-napplicable.

## Data Availability

The datasets can be made available upon reasonable request for scientific collaborations. Further information, including the procedures to obtain and access data, can be obtained from the corresponding author upon request.

## Abbreviations

BMFI: Body mass fat index
BMI: Body mass index
BWRP: Body weight reduction program
DBP: Diastolic blood pressure
FFM: Fat-free mass
FM: Fat mass
HDL-C: High-density lipoprotein cholesterol
MetS-: Negative for metabolic syndrome
MetS+: Positive for metabolic syndrome
SBP: Systolic blood pressure
sd: Standard deviation
yrs: Years
WC: Waist circumference.
